# Genome-Based Taxonomy of Species in the *Pseudomonas syringae* and *Pseudomonas lutea* Phylogenetic Groups and Proposal of *Pseudomonas maioricensis* sp. *nov.,* Isolated from Agricultural Soil

**DOI:** 10.3390/microorganisms12030460

**Published:** 2024-02-24

**Authors:** Magdalena Mulet, Margarita Gomila, Antonio Busquets, David Sánchez, Jorge Lalucat, Elena García-Valdés

**Affiliations:** Microbiología (Biology Department), Universitat de les Illes Balears, 07122 Palma de Mallorca, Spain; mmagdalena.mulet@uib.es (M.M.); marga.gomila@uib.es (M.G.); toni.busquets@uib.es (A.B.); dsanzber@gmail.com (D.S.)

**Keywords:** taxonomy, phylogenomics, *Pseudomonas syringae*, *Pseudomonas maioricensis*

## Abstract

Species in the phylogenetic group *Pseudomonas syringae* are considered the most relevant plant pathogenic bacteria, but their taxonomy is still controversial. Twenty named species are validated in the current taxonomy of this group and in recent years many strains have been genome-sequenced, putative new species have been proposed and an update in the taxonomy is needed. A taxonomic study based on the core-genome phylogeny, genomic indices (ANI and GGDC) and gene content (phyletic pattern and Jaccard index) have been applied to clarify the taxonomy of the group. A phylogenomic analysis demonstrates that at least 50 phylogenomic species can be delineated within the group and that many strains whose genomes have been deposited in the databases are not correctly classified at the species level. Other species names, like “*Pseudomonas coronafaciens*”, have been proposed but are not validated yet. One of the putative new species is taxonomically described, and the name *Pseudomonas maioricensis* sp. nov. is proposed. The taxonomies of *Pseudomonas avellanae* and *Pseudomonas viridiflava* are discussed in detail as case studies. Correct strain identification is a prerequisite for many studies, and therefore, criteria are given to facilitate identification.

## 1. Introduction

The genus *Pseudomonas* encompasses a large number of species, with more than 270 named species in the current taxonomy, as stated in the List of Prokaryotic Names with Standing in Nomenclature [1] (https://www.bacterio.net/, last accessed in 22 October 2023). *Pseudomonas* species are ubiquitous, but some are preferentially present in the environment, including soil (*Pseudomonas putida* group) or fresh water (*Pseudomonas fluorescens* group); others are known to be pathogenic to humans (*Pseudomonas aeruginosa* group) or insects (*Pseudomonas entomophila*), and finally, some are related to plants, including those that are beneficial (*Pseudomonas protegens* group) and those that are pathogens (*Pseudomonas syringae* group) [2]. Pseudomonads can cause diseases in an array of plant species [3,4,5], and *P. syringae* is considered the top species within the top 10 plant pathogenic bacteria [6]; however, its taxonomy is controversial, and many strains are included in the so-called *P. syringae* species complex, which is phenotypically divided into more than 60 pathovars that can cause diseases in monocots, dicots and woody plants [7]. Pathovars are not always supported by phylogenetic traits, but at least a total of thirteen phylogenetic groups have been devised based on the similarity of housekeeping genes [8].

Several phylogenetic groups of species can be distinguished in the genus *Pseudomonas* [2], and most of the plant pathogenic species are included in the *P. syringae* phylogenetic group, comprising the *P. syringae* species complex and the named *Pseudomonas* species, including *P. amygdali* (which includes *P. meliae*, *P. ficuserectae* and *P. savastanoi*), *P. asturiensis*, *P. avellanae*, *P. cannabina*, *P. caspiana*, *P. caricapapayae, P. cerasi*, *P. cichorii*, *P. congelans*, *P. syringae*, *P. tremae* and *P. viridiflava*, as described by Gomila et al. in 2017 [9]. Since that publication, nine new species have been described in the group (*P. alliivorans*, *P. capsici*, *P. floridensis*, *P. folii*, *P. lijiangensis*, *P. quasicaspiana*, *P. triticifolii* and *P. ovata*). “*P. coronafaciens*” also belongs to the group, but the name has not been validated. Numerous strains have been genome-sequenced, and putative new species have been proposed, highlighting the necessity for an updated taxonomy because genomic analysis has profoundly changed the taxonomy of the genus [2,10]. With this objective in mind, a genome-based taxonomic study was carried out, including species in the closely related phylogenetic group of *Pseudomonas lutea* that includes the following *Pseudomonas* species: *P. abietaniphila*, *P. bohemica*, *P. graminis*, *P. lutea* and *P. petrae*. One of the new putative species, represented by strain S25T, is phenotypically, chemotaxonomically and genomically described to propose *Pseudomonas maioricensis* sp. nov., with strain S25T (= CCUG 69272T, CECT 30911T) as the type strain.

## 2. Materials and Methods

### 2.1. Pseudomonas Strains and Growth Conditions

The strains studied are detailed in Table 1, which includes all type strains of the *P. syringae* and *P. lutea* groups, the related reference strains considered in the GTDB taxonomy, and other *Pseudomonas* strains not classified at the species level or incorrectly classified and whose genomes are available in the NCBI database (https://www.ncbi.nlm.nih.gov/, last visited in 22 October 2023). Two strains considered to be putative new species in previous publications were included in the analysis: strains S25T [9] and JDS28PS106 [11]. Strain S25T was isolated in September 2010 from an agricultural soil sample collected at the campus of the University of the Balearic Islands in Mallorca, Spain (39°38′11.8″ N, 2°38′50.1″ E), during a screening for environmental bacteria. From a suspension of 2 g of soil in 8 mL of Ringer (Merck), 100 µL was plated on Middlebrook agar supplemented with Middlebrook Oleic Albumin Dextrose Catalase Growth Supplement (Middlebrook OADC enrichment, Difco, Madrid, Spain) under aerobic conditions. The plates were incubated at 30 °C for four days. Colonies were checked for purity on agar-solidified LB medium (lysis broth) [12]. Strain JDS28PS106 was isolated from a water sample taken from the Danube River and proposed as the new phylospecies PS8 in a multilocus sequence analysis [11]. Bacteria were cultured routinely at 30 °C on LB media.

### 2.2. Genome Sequencing

Genomic DNA was isolated from the S25T and JDS28PS106 strains using a Wizard Genomic DNA Purification Kit (Promega) according to the manufacturer’s instructions. The paired-end library reads obtained from the Illumina HiSeq 2000 platform were assembled de novo using the Newbler Assembler v2.7 program (Roche). The draft genomes were annotated using the NCBI Prokaryotic Genome Annotation Pipeline (PGAP). The whole-genome shotgun sequencing data of strains S25T and JDS28PS106 have been deposited into DDBJ/EMBL/GenBank under the accession numbers LOHG00000000.1 (BioProject PRJNA305113) and JBAJGT000000000 (BioProject PRJNA1048145), respectively.

### 2.3. Phylogenomic Analysis

The genomic relatedness of all species type strains, GTDB reference strains and closely related strains in the *P. syringae* and *P. lutea* phylogenetic groups of publicly available species (comprising a total of 74 genomes) was determined based on the genome-aggregated average nucleotide identity (ANI) determined with the BLAST algorithm. It was calculated using the JSpecies software tool accessible at http://jspecies.ribohost.com/jspeciesws/ (last accessed in 22 October 2023) [13]. Additionally, digital DNA–DNA hybridisation between the selected strains was performed by the genome-to-genome distance (GGDC) method. The GGDC was calculated using the web service http://ggdc.dsmz.de, last accessed in 22 October 2023 [14], and the recommended BLAST method. The GGDC results presented herein were based on the recommended Formula 2. The similarity of the genomes of closely related species-type strains was also calculated using the Type Strain Genome Server (TYGS), a free bioinformatics platform (https://tygs.dsmz.de, accessed on 22 October 2023) [15]. The recommended thresholds considered for species differentiation are 95–96% for ANI [13] and 70% for GGDC [14].

The M1CR0B1AL1Z3R web server (https://microbializer.tau.ac.il/, last accessed in 22 October 2023) [16] was used to identify orthologous genes in the genomes. Briefly, the server extracts the orthologous sets of genes in each genome and analyses the gene presence–absence patterns. The settings applied were as follows: maximal e-value cut-off: 0.01; percent identity cut-off: 70.0%; and minimal percentage for the core: 100.0%. The concatenated sequences of the resulting core proteomes were used to infer the phylogeny of the strains, and the results were visualised by the server in a RAxML tree. Two strains were considered members of the same phylogenomic species (pgs) when both were affiliated with the same phylogenetic branch, and the average ANI and GGDC values were above the established species thresholds.

The Jaccard similarity index implemented in the PAST package of programs [17] was used as a measure of similarity in pairwise comparisons. The Jaccard index of similarity was calculated as $SJ=\frac{a}{a+b+c+d}$, in which a is the number of genes that were present in both genomes of each pair, b and c are the number of genes present in one strain but absent in the other, and d is the number of orthologues in the group of strains studied that are absent in both of the compared strains. The final matrix was represented in a UPGMA dendrogram with PAST [17]. The orthologous genes shared by the pairs of strains were also represented in a split-tree decomposition. SplitsTree software (version 5) was used for computing unrooted phylogenetic networks from molecular sequence data, as discussed by Huson and Bryant [18]. The phyletic pattern obtained with M1CR0B1AL1Z3R, that is, the presence or absence of the orthologous genes in each strain, was also represented in a heat plot generated using the PAST program. A Venn diagram was constructed on the web page https://bioinfogp.cnb.csic.es/tools/venny/ last accessed on 22 October 2023 [19] to differentiate the closely related strains in the *P. caspiana* phylogenetic branch and to identify exclusive and shared genes. Individual genes were identified by BLAST in the NCBI database (https://www.ncbi.nlm.nih.gov/, last accessed on 22 October 2023) and in the *Pseudomonas* genome database (https://www.pseudomonas.com/, last accessed on 22 October 2023) [20] or were annotated with Rapid Annotation using Subsystems Technology (RAST; https://rast.nmpdr.org/, last accessed on 22 October 2023) [21]. Phylogenies were also constructed with the autoMLST website [22] as previously reported [2] and were also retrieved from the GTDB website (https://gtdb.ecogenomic.org/, last accessed in 22 October 2023) [23].

### 2.4. Genomic Insights

The following databases were used to detect diagnostic or relevant genes related to the taxonomy of the new species according to the S25T genome: the NCBI; the Kyoto Encyclopedia of Genes and Genomes (KEGG; https://www.genome.jp/kegg/, last accessed in 22 October 2023) [24]; the Virulence Factors of Pathogenic Bacteria database (http://www.mgc.ac.cn/VFs/, last accessed in 22 October 2023) [25]; the *P. syringae* Genome Database (http://www.pseudomonas-syringae.org/, last accessed in 22 October 2023); the PHASTER (PHAge Search Tool Enhanced Release) web server for the identification and annotation of prophage sequences within bacterial genomes (http://phaster.ca/, last accessed in 22 October 2023) [26]; and the toxin–antitoxin Finder (TA Finder; http://202.120.12.133/TAfinder/index.php, last accessed in 22 October 2023). If not otherwise stated, a gene was considered present following the 50/50 criterion (similarity higher than 50% in at least 50% of the sequence coverage) with a gene in the database. The predicted functional categories and COG classifications were established via protein BLAST against all coding gene sequences in the COG database available at NCBI. Antibiotic resistance genes were identified in the genome with the Comprehensive Antibiotic Resistance Database (CARD) [27].

### 2.5. Characterisation of Strain S25T

Cell size, morphology and flagellar insertion were determined via the transmission electron microscopy of cells from the exponential growth phase in LB. A Talos F200i electron microscope (Thermo Fisher, Barcelona, Spain) was used at 80 kV. The samples were negatively stained with uranyl acetate in aqueous solution (2%) as described by Lalucat [28]. The presence of fluorescent pigments was tested on King B media (Pseudomonas agar F, Difco, Madrid, Spain), and pyocyanin production was tested on King A media (Pseudomonas agar P, Difco). The S25T strain and other type strains in the study were characterised phenotypically using API 20 NE strips (bioMérieux) and Biolog GN2 MicroPlates (Biolog, Hayward, CA, USA). Plant pathogenicity tests of the strain S25T were performed on citrus leaves as described by Beiki and collaborators [29]. Briefly, the strains were cultivated on nutrient agar at 25 °C for 24 h, after which the cells were suspended in sterile distilled water at approximately 1 × 10^8^ CFU/mL. One hundred microlitres of solution was injected into the intercellular space of the orange leaves with a 0.5 mm needle, after which the plants were incubated at 20 °C. Symptoms were observed for one week.

To determine the whole-cell protein profile, a matrix-assisted laser desorption/ionisation time-of-flight mass spectrometry (MALDI-TOF MS) analysis of the S25T strain was performed, together with its closely related strains, at the Scientific-Technical Services of the University of Balearic Islands (Spain) as described by Sanchez et al. [30]. The profile obtained for each species was analysed and compared, and the corresponding dendrogram was generated using MALDI BioTyper software (version 1.0; Bruker Daltonics).

Whole-cell fatty acid methyl ester (FAME) analysis was performed at the Spanish Type Culture Collection (CECT), Valencia, Spain. The strains were cultured for 24 h in Trypticase Soy Agar at 28 °C. Fatty acids were extracted and prepared according to standard protocols as described for the MIDI Microbial Identification System [31]. The cellular fatty acid content was analysed via gas chromatography with an Agilent 6850 unit, the MIDI Microbial Identification System using the RTSBA6 method [32] and the Microbial Identification Sherlock software package version 6.1.

Experimental DNA-DNA hybridisation was performed as previously described [30].

## 3. Results

### 3.1. Phylogenomic Analysis

**Figure 1:**
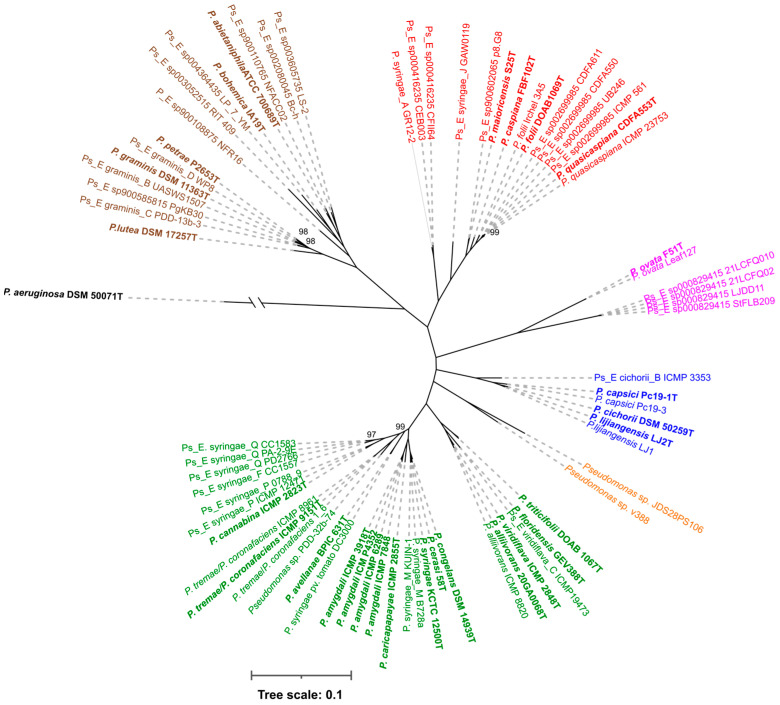
RAxML phylogenetic tree (1505 core genes) of representative strains in the *P. syringae* and *P. lutea* phylogenetic groups with *P. aeruginosa* as the outgroup. The numbers in the nodes are bootstrap values of 100 replicates. Main phylogenetic branches are labelled in different colours.

**Figure 2:**
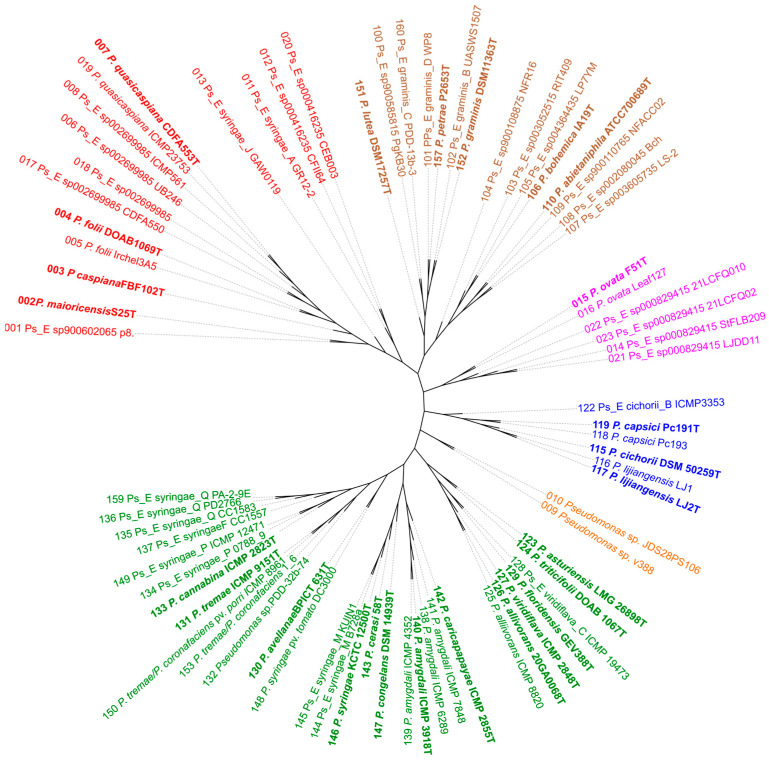
RAxML phylogenetic tree (2015 genes of the core proteome) of representative strains in the *P. syringae* and *P. lutea* phylogenetic groups without an outgroup. Main phylogenetic branches are labelled in different colours.

The core proteome was calculated with the M1CR0B1AL1Z3R web server for all the genomes using the *P. aeruginosa* type strain as an outgroup. The concatenated gene sequences of the 1505 core genes (a total core length of 552,494 nt) allowed us to infer the phylogenetic tree depicted in Figure 1 with RAxML. All branches were supported by high bootstrap values (95 of the values were 100, and only 4 values were 97–99 based on 100 replicates). The strains were clustered into five main branches represented by *P. syringae*, *P. cichorii*, *P. ovata*, *P. caspiana* and *P. lutea*. Two strains, v388 and JDS28PS106, were located on a different branch. The OWCr strain classified as *P. caspiana* was clearly outside these groups belonged to the *P. mandelii* subgroup of the fluorescens group and was not further studied. To obtain a greater number of orthologous genes shared by each pair of strains, the analysis was also repeated without an outgroup. The core proteome consisted of 2015 genes (a total core length of 727,877 nt), and the main branches were maintained as depicted in Figure 2. Clustering was in accordance with the GTDB taxonomy, the autoMLST phylogeny and previous studies, but the branching order was slightly different. The *P. lutea* and *P. ovata* branches were located between the *P. syringae* and the *P. caspiana* branches when the tree was constructed without an outgroup.

**Figure 3:**
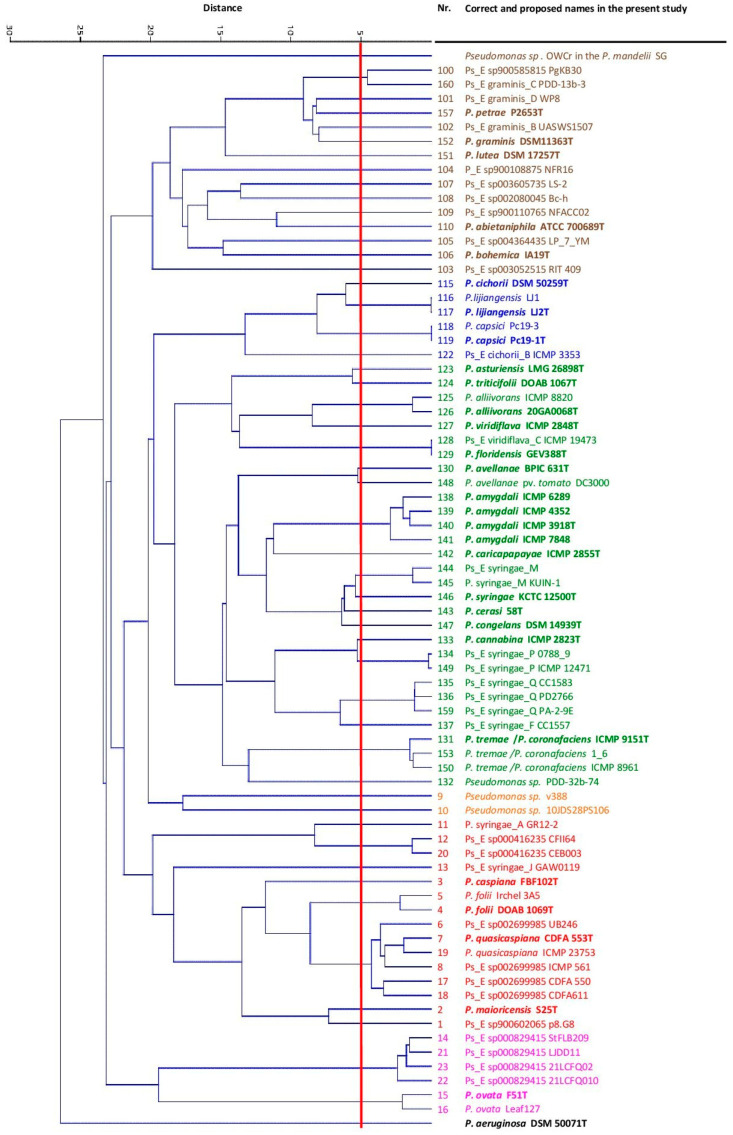
Dendrogram of the ANI values among the strains studied. Type strains are highlighted in bold. Main phylogenetic branches are labelled in different colours. Red line: species threshold. Scale: ANI distance.

The ANI in the pairwise comparisons was calculated for all the genomes. The matrix is represented in the corresponding UPGMA dendrogram in Figure 3. The main branches were maintained. In total, 50 phylogenomic species could be differentiated. *P. meliae*, *P. savastanoi* and *P. ficuserectae* belonged to the *P. amygdalii* phylogenomic species that was previously reported. The five putative novel species proposed in a previous publication [9], species A, B, C, D and E, were confirmed to be different pgs. In the present study, species A was included in Pseudomonas_E syringae_M (strain B728a), B was included in Pseudomonas_E syringae_Q (strain CC1583), C was included in *P. triticifolii* (strains CC1417 and CC1524), species D was included in Pseudomonas_E sp002699985 (strain UB246) and E was included in the newly proposed species *P. maioricensis* (strain S25). Twenty-two of the 50 pgs defined are currently named species; the rest are representatives of potential novel species and were mainly singletons. A high percentage of the strains whose genomes have been deposited in the NCBI database are not correctly classified at the species level in the current taxonomy, as indicated in Table 1, and will be discussed later. For example, 13 *P. syringae* strains were phylogenetically related to 10 different pgs, some of which were in the *P. caspiana* phylogenetic branch. Additionally, 17 deposited genomes from strains not identified at the species level corresponded to 13 potential new species.

Notably, the species *P. avellanae* was one of the most abundant. The GTDB taxonomy includes 353 strains in the species cluster, but only 17 are classified in the NCBI taxonomy as *P. avellanae*. The other strains, including pathovar reference strains, were assigned to different species: 310 in *P. syringae*, 20 in *P. viridiflava*, and 6 in *P. amygdali*, reflecting difficulty in species identification. A detailed study of 44 *P. avellanae* strains by M1CR0B1AL1Z3R, ANI and GGDC (Supplemental Figure S1) revealed that two branches are phylogenetically differentiated. One group was represented by *P. avellanae* BPIC631T, and the other was represented by *P. syringae* pv. *tomato* DC3000. The ANI and GGDC values among members of both groups were less than 96% ANI and less than 70% GGDC; the intragroup values were higher than 96,5% ANI and 78% GGDC in the *P. avellanae* group and higher than 98% ANI and 90% GGDC in the DC3000 group.

The strains analysed that were assigned to “*P. coronafaciens*” in the NCBI taxonomy were classified as *P. tremae* in the present study and in the GTDB taxonomy. Therefore, additional strains classified as “*P. coronafaciens*” in the NCBI taxonomy were analysed on the autoMLST web server, together with the *P. tremae* and “*P. coronafaciens*” type strains. The results are given in Supplementary Figure S2. Both species are monophyletic, and the ANI indices among all the strains are greater than 98%. Additionally, these species must be considered members of the same phylogenomic species.

### 3.2. P. viridiflava Case Study

One thousand five hundred and twenty-two genome-sequenced strains of the Bioproject PRJEB24450 assigned taxonomically to the species *P. viridiflava* [33] were analysed in the present study to test the superiority of the phylogenomic approach in the current taxonomy. The phylogeny was inferred using the GTDB and the AutoMLST website. Most sequences from the RefSeq collection in the NCBI database were suppressed because of an unverified organism source, although the isolate identities were reported on the web site and in the original publication [33]; therefore, they were included in our study. As indicated in Table 2, our results demonstrated that most of the strains (90%) were correctly assigned to *P. viridiflava,* but 164 (10%) belonged to 37 different pgs (15 named species and 22 putative new species). Eleven pgs were phylogenetically placed in the *P. lutea*/*P. syringae* groups (43 strains), but the majority of the strains belonged to the *P. fluorescens* group or were located close to other phylogenetic groups (Table 2).

### 3.3. Gene Content Comparisons

**Figure 4:**
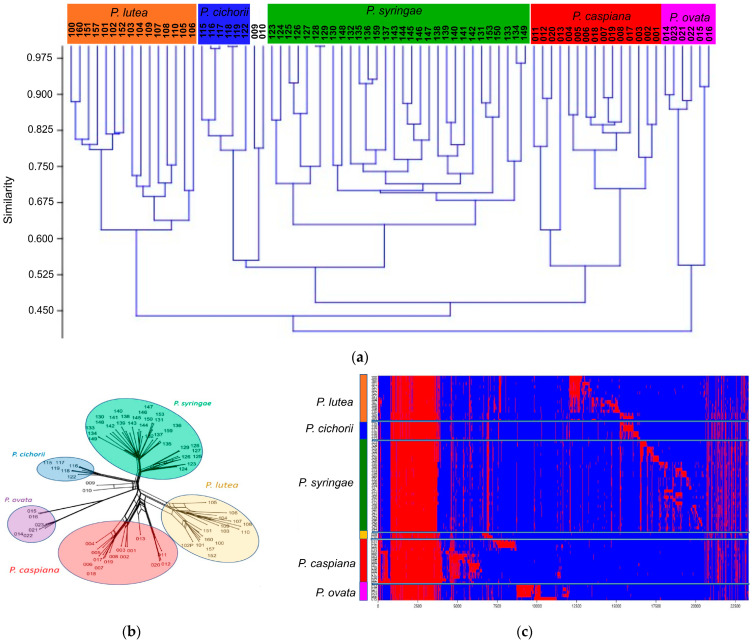
Gene content distribution of the 23,319 orthologues calculated by M1CR0B1AL1Z3R, represented by the Jaccard index (**a**) (discontinuous red line indicates the 6 main phylogenetic branches), a split-tree decomposition (**b**) and a heatmap (**c**). Each phylogenetic branch is highlighted with a different colour. Numbers indicate the strain in Table 1.

The core proteomes of the 74 genomes in the *P. syringae* and *P. lutea* groups calculated by M1CR0B1AL1Z3R consisted of 2015 genes of the 23,319 orthologues. The genes shared by each pair of strains were studied in three different ways: the Jaccard index, split tree decomposition and heatmap analysis. The grouping of strains by the three methods confirmed the main phylogenetic branches defined by the core proteome and are depicted in Figure 4a–c (Jaccard, Splits and heatmap, respectively). The strains in each cluster were at least 0.55 similar according to the Jaccard index. The presence/absence of the 23,319 orthologous genes in each strain is clearly presented in the matrix plot (Figure 4c heatmap). A total of 2015 core genes were present in all the strains, and the others were preferentially distributed within the phylogenetic branch; however, it was not possible to detect a significant group of genes that were present in all the strains in a branch and that were absent in the other groups.

### 3.4. Characterisation of P. maioricensis S25T sp. nov.

Strain S25T was clearly representative of a phylogenomic species that is different from the others; therefore, it was taxonomically described. Strain S25T was proposed in the present study as the type strain of a novel species, *Pseudomonas maioricensis*. The closest type strain species identified in the ANI and GGDC analyses were *P. caspiana* (86% ANI and 32.7% GGDC) and *P. quasicaspiana* (88 ANI and 33.4 GGDC). Experimental DNA—DNA hybridisation (DDH) was performed to confirm the genomic differentiation of strain S25T from *P. caspiana* strains. The labelled DNA of strain S25T and *P. caspiana* FBF102T strain was hybridised separately with DNA from selected type strains of the *P. syringae* phylogenetic group and from other *P. caspiana* strain. The labelled DNA of the S25T and FBF102T strains presented DDH values less than 60%, with all the type strains tested, and did not meet the 70% threshold established for species DNA–DNA similarity. The DDH values between S25T and FBF102T were less than 50%. These experimental results confirmed the results of the digital DNA–DNA hybridisation: strain S25T is not a member of any other *Pseudomonas* species tested (Table S1).

#### 3.4.1. Genome Insights

**Figure 5:**
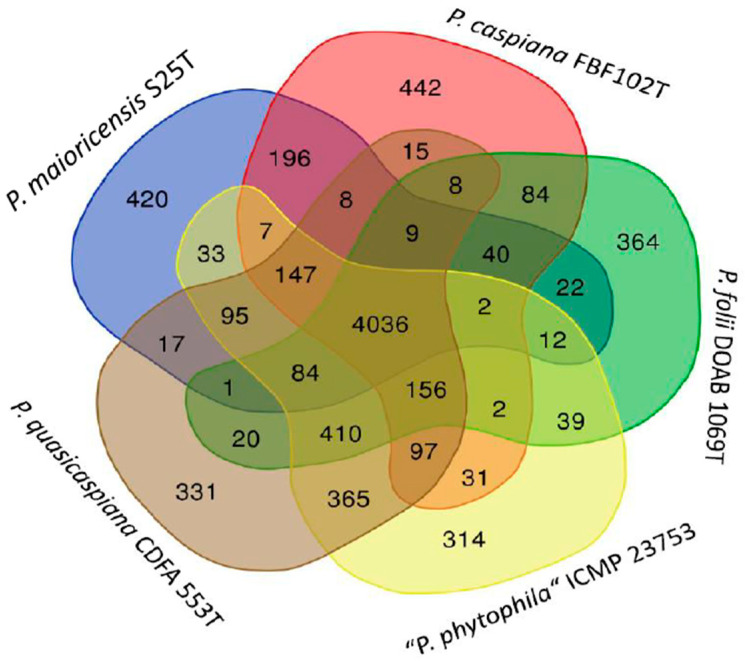
Venn diagram of the orthologous genes shared by the strains studied in the *P. caspiana* branch.

The gene content of strain S25T was studied in detail by comparing this strain with four other selected strains in the *P. caspiana* branch, including the three species type strains and the strain proposed as “*P. phytophila*”. The analysis performed with the M1CR0B1AL1Z3R web server identified 4018 genes shared by all five strains and a significant number of exclusive genes (called “orphan genes” or singletons) that were not shared with any other strain in the study (Table S2). The 281 exclusive genes of strain S25T were analysed manually. Those containing a high number of unresolved nucleotides (N) were discarded, and the rest of the sequences were concatenated and annotated with RAST. The results are summarised in Table S3. Two hundred and twenty-five proteins were annotated as hypothetical proteins; 43 were assigned a function, and 5 were predicted to be phage proteins. The orthologous genes shared among the five selected strains are represented in a Venn diagram (Figure 5). The exclusive genes of each strain in the diagram are orthologues also present in some other strains in the set of 74 genomes studied in the *P. syringae*-*P. lutea* phylogenetic groups.

The analysis performed with the KEGG website allowed us to infer the metabolic characteristics of strain S25T. No significant differences could be found between strain S25T and the other four closely related strains included in the study. All the strains contained enzymes for a functional Entner–Doudoroff pathway for the catabolism of sugars, for starch hydrolysis, for the ortho cleavage pathway, for the degradation of aromatics, and for the reduction of nitrate to ammonia but not for denitrification. The five strains had secretion systems of types 2 and 6 (T2SS and T6SS).

To identify strain- or species-specific genes, a prophage survey was performed on the PHASTER server. As shown in Table S4, no complete prophage was found in strain S25T, although two incomplete phages were detected (9.8 Kb—7 proteins; and 8.1 Kb—9 proteins). No prophages were found in the genomes of *P. caspiana* FBF102T or *P. quasicaspiana* CDFA 553T. A region with a complete phage with 49 proteins was found in *P. folii* DOAB 1069T, and two intact and two incomplete phages were found in “*P. phytophila*” ICMP 23753.

Given the importance of the strains of this group as potential plant pathogens, the genes involved in pathogenesis were studied in detail. Supplementary Table S5 summarises the relevant genes found in the genome of strain S25T: (i) a complete set of genes for flagellation and chemotaxis; (ii) essential genes for pyoverdin synthesis; and (iii) genes involved in adherence, such as a complete set of alginate genes (regulation, polymerisation, acetylation, epimerisation and the export of alginate), and type IV pili synthesis with a role in biofilm formation; and (iv) genes for antimicrobial activities that can confer a competitive advantage. Several efflux pumps related to fluoroquinolone transport were found and are listed in Table S6. The AcrAB genes encode the acriflavine resistance protein and, together with TolC, constitute a tripartite complex of an efflux pump that may mediate resistance against host-derived antimicrobial peptides and is associated with antibiotic resistance (Table S6). At least three types of possible toxin and antitoxin systems were found using the KEGG website: HigA/HigB, HigA-1/HigB-1 and mqsA/mqsR.

**Figure 6:**
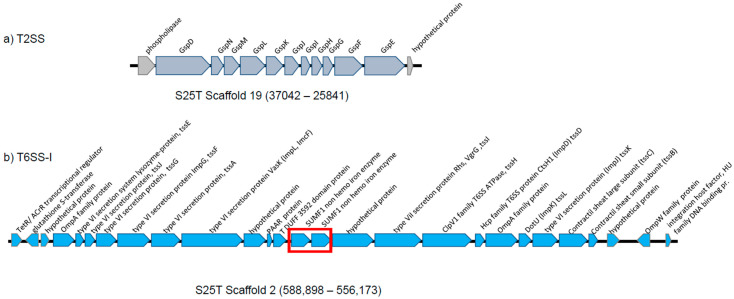
Organisation of the *P. maioricensis* S25T T2SS (**a**) and T6SS-I (**b**) clusters and flanking regions. Arrows indicate the detected ORFs and their direction of transcription. The red square indicates a possible gene duplication in *P. maioricensis* S25T that was absent in *P. caspiana* FB102T.

Secretion systems are crucial for bacterial pathogenicity in plants. Those present in strain S25T were compared to those present in *P. caspiana* FBF102T, the closest relative whose pathogenicity has been proven [34]. The S25T strain has complete sets of genes for the synthesis of type 1, 2 and 6 secretion systems (TSSs). The gene organisation of T2SS was identical to that of the corresponding system in *P. caspiana* (Figure 6a). Three variants of the T6SS are known, two of which have been recognised in *P. caspiana* FBF102T. Only one T6SS was found in strain S25T, which corresponded to T6SS-I and contained 27 CDSs, while the *P. caspiana* FBF102T TSS-I contained 26 (Figure 6b). The FBF102T coding sequence AUC60_24260 has two putative homologues in the S25T genome: AUC61_05175 (99% coverage and 69% identity) and AUC61_05170 (95% coverage and 73% identity). These two CDSs are tandem and 70% similar, with a coverage of 97%, probably resulting from a duplication process. The 32,725-nucleotide-long fragment of scaffold 2 of S25T (positions 588,898–556,173) had 98% (84% identity) coverage with the *P. caspiana* FBF102T scaffold 30 (1–32,820 bp) (Figure 6b and Table S7). Several VgrG alleles of the S25T strain and structural and core components of T6SS were found: PROKKA_01053 (93,9% identity with *P. caspiana* FBF102T), PROKKA_01334 (91,90% identity with *P. viridiflava*), PROKKA_01488 (91,52% identity with *P. quasicaspiana*), PROKKA_02799 (90% identity with putative new species *Pseudomonas* sp. ICMP 561) and PROKKA 04,953 (78,35% identity with *P. viridiflava*). No T6SS-II type has been found in strain S25T, although it has been reported in the phylogenetically closest species *P. caspiana* FBF102T [34].

#### 3.4.2. Cell Morphology, Physiology and Biochemical Characterisation

The S25T strain is a Gram-negative, rod-shaped bacterium with polar flagellation, as shown by the electron microscopy images in Figure S3. After incubating for 48 h at 30 °C, the colonies were round (1–1.5 mm in diameter), flat, beige coloured, had regular margins and were translucent. The S25T strain was positive for catalase and oxidase activities, was strictly oxidative (not fermentative) and was not a denitrifier. The S25T strain was able to grow in LB media at 4–30 °C for 24–48 h after one week of incubation. No growth was detected at 42 °C or at 37 °C. Growth was observed on nutrient broth in the presence of NaCl until a concentration of 6% (*w*/*v*) was reached and the tolerated pH ranged from 5 to 9. Strain S25T showed fluorescent pigmentation on Pseudomonas agar F and did not produce pyocyanin on Pseudomonas agar P when cultured for 24–48 h at 30 °C. A complete list of the results is given in Table S8. Strain S25T was not pathogenic to the citrus plants according to the tests performed, as shown in Supplementary Figure S4. The relevant differential characteristics of strain S25T are given in Table 3 and in the protologue Table 4.

#### 3.4.3. Chemotaxonomic Analysis

The dendrogram obtained by WC-MALDI-TOF MS analysis showed that the S25T strain, *P. quasicaspiana* strains and *P. foli* strains clustered together at 70%. The similarity of the strains in the *P. caspiana* and *P. syringae* groups was lower than 45% (Figure S5). The S25T strain was located on an independent branch of the *P. caspiana* strains at a 10% distance, so that it could be easily differentiated in the dendrogram.

The fatty acid profiles of *P. caspiana* FBF102 T, FBF122 and S25T were similar (Table S9). The results were also very similar to the characteristic fatty acid profiles of the *P. syringae* group. The major cellular fatty acids in strain S25T were C10:0 3-OH (3.0%), C12:0 (6.3%), C12:0 2-OH (2.7%), C12:0 3-OH (5.3%), C16:0 (24.6%), C18:0 (1.5%), the summed feature 3 C16:1 w7c/C16:1 w6c (342%) and the summed feature 8 C18:1 w7c/C18:1 w6c (18.8%). C17:0 cyclo was present in low amounts (1.9%). C17:0 cyclo was absent in most of the species of the *P. syringae* group, except for *P. asturiensis* and *P. ficuserectae* [35].

## 4. Discussion

The value of the genomic approach in the current taxonomy of species within the *P. syringae* and *P. lutea* phylogenetic groups is highlighted in the present study. All the data presented demonstrate phylogenetic consistency and clear genomic indices for species differentiation. At least ten not-yet-named novel phylogenomic species could be delineated in the *P. lutea* group, six in the *P. syringae* branch, five in the *P. caspiana* branch, two in the *P. cichorii* branch and one in the *P. ovata* branch. The other two pgs (v388 and JDS28PS106) were not affiliated with any of the branches. This substantial increase in the number of species within the group will undoubtedly hinder species identification via routine analysis. These 26 potential novel species have been misidentified thus far or have been classified only at the genus level. It is also important to emphasise that the named species *P. meliae*, *P. savastanoi* and *P. ficuserectae* are synonyms of *P. amygdali*. This result was already published by Gardan et al. in 1999 after experimental DNA–DNA hybridisations that grouped the four species type strains into the same genomospecies [36]. The genome sequence of strain ICMP 23,753 was deposited in the NCBI as “*P. phytophila*”, but the name has never been formally proposed, and the present study demonstrated that it belongs to the species *P. quasicaspiana*.

However, the classification of the important pathovar reference strain *P. syringae* pv. tomato DC3000 is controversial [37]. It is a model plant pathogen that causes disease in tomato and in the model plants *Arabidopsis thaliana* and *Nicotiana benthamiana*. Strain DC3000 does not taxonomically belong to *P. syringae*. The GTDB taxonomy includes DC3000 in *P. avellanae*; however, the *P. avellanae* strains are divided into two clear phylogenetic groups separated by the ANI and GGDC indices: the 96% ANI and 70% GGDC thresholds. As proposed in a previous publication [9], the group represented by strain DC3000 could be classified into a different species, subspecies or new genomovar; however, a definitive taxonomic status requires additional investigations and a formal taxonomical proposal. As stated by Scortichini et al. [37], “Name changes may be confounding and should be attempted with caution”.

This challenge in identification is exemplified in the important study on the population dynamics of *P. viridiflava* developed by Karasov et al. [33], in which 1522 strains were initially grouped by their 16S RNA sequence and identified as *P. viridiflava*; however, the GTDB phylogenomic identification in the present study assigned 10% of them to 15 named species and 22 pgs that require further characterisation.

Notably, “*P. coronafaciens*” is not included in the List of Bacterial Names with Standing in the Nomenclature. The species was initially proposed by Schaad and Cunfer [38] but was not included in the Approved Lists of Bacterial Names [39]. Dutta et al. [40] proposed the revival of the name “*P. coronafaciens*”, but the genome sequence of the proposed type strain (NCPPB 600T = CFBP 2216T = LMG 5060T = ICMP 3316T) was not included in their analysis. The genome sequence of “*P. coronafaciens*” LMG 5060T (GCA_000773135.1) together with 76 other genomes of “*P. coronafaciens*” strains are available on the NCBI website, but these strains are classified as *P. tremae* in the GTDB taxonomy because the type strain *P. tremae* ICMP 9151T is included in the cluster. “*P. coronafaciens*” is not considered in the GTDB taxonomy. The “*P. coronafaciens*” LMG 5060T genome remains “undefined” in the GTDB taxonomy because the sequence failed the quality check (1981 contigs). Additionally, in a previous publication [9], the genome sequence of the *P. tremae* ICMP 9151T strain was revised due to inconsistencies in the multilocus sequence analysis, and the genome failed the quality analysis with the GTDB taxonomy. In our phylogenomic study, the strains “*P. coronafaciens*” LMG 5060T and *P. tremae* ICMP 9151T were identified in the same species, as well as in other *P. coronafaciens* and *P. tremae* strains. However, further genomic studies are needed to determine whether the name “*P. coronafaciens*” has to be revived or considered synonymous with *P. tremae*.

Routine biochemical tests often fail to differentiate many species in the so-called *P. syringae* complex [41]. Consequently, molecular identification techniques have become essential, and phylogenomic classification is currently considered the most effective method. Simultaneously, genomic techniques allow us to at least partially justify the enormous phenotypic diversity observed within the group. In this context, the study presented highlights the considerable number of genes present in the strains of the group (pangenome). Additionally, this study revealed how these genes can be shared and exchanged among strains. Despite this genomic fluidity, the combination of specific genes defines the distinct phylogenetic branches and, by extension, the individual species within the complex.

As an example of the use of the proposed phylogenomic approach in the description of new *Pseudomonas* species, we have presented the characterisation of the strain S25T. *Pseudomonas* sp. S25T was initially described as a putative new species in the *P. syringae* phylogenetic group of species in 2017 [9]. The differentiation of this species from its closest relatives was confirmed in the present study by new molecular and phenotypic data from a polyphasic perspective. Its phenotypic characteristics are corroborated in part by genomic insights, as well as by the potential for its presence in the genome or phage genes for strain differentiation. An interesting point is the existence of a T6SS that is used by many Gram-negative bacteria to deliver toxins that seem to confer competitive advantages in both environmental and pathogenic bacteria and are present in the majority of plant pathogens [42]. It cannot be ruled out that the T6SS has other effects in addition to pathogenicity since many plant-growth-promoting bacteria also possess one or more T6SSs [42]. A single strain may harbour multiple T6SSs [34,42], such as *P. caspiana* FBF102T, the closest plant pathogen relative to S25T. *P. maioricensis* S25T possesses a single T6SS with a small difference (a gene duplication) with respect to one of the two T6SSs of *P. caspiana* FBF102T, but S25T is not pathogenic according to laboratory tests. It is possible that both subtypes of the T6SS are necessary or that gene duplication may have a negative effect. However, the role of the T6SS in S25T needs further experimental investigation.

The rapid increase in the number of *Pseudomonas* species described in recent years presents a significant challenge in the routine identification of new isolates, both pathogenic and environmental saprophytic. Therefore, it is necessary to maintain an updated taxonomy to facilitate accurate identification. The present study serves to clarify the classification and provides identification criteria for species delineation within one of the most important groups of plant pathogenic bacteria. The sequencing of genomes and selected genes, such as the *rpoD* gene, in combination with phenotypic identification based on whole-cell protein profiles obtained by MALDI-TOF MS is currently recognised as the most valuable taxonomic tools.

## 5. Description of *Pseudomonas maioricensis* sp. nov.

The morphological, physiological, biochemical, chemotaxonomic, phylogenetic and genomic characteristics support the proposal of a new bacterial species based on the type strain S25T (= CCUG 69272T, CECT 30911T). The complete description is given in the protologue presented in Table 4.

## Figures and Tables

**Table 1:** List of bacterial strains and genome accession numbers, NCBI identification, GTDB classification and proposed identification in the present study. Nr: strain number in the present study. Species type strains are labelled in bold. Ps_E: abbreviation of the genus Pseudomonas_E in the GTDB taxonomy.

Nr	Strain	NCBI Name	Assembly	GTDB Taxonomy			This Study
				Proposed species name	Representative genome *	Strains in species cluster	Correct and proposed names in the present study
**--**	**DSM 50071T**	***P. aeruginosa***	**GCF_001045685.1**	***P. aeruginosa***	**false**	**7037**	***P. aeruginosa***
--	OWCr	*P. caspiana*	GCF_006439035.1	Ps_E mandelii_C	false	4	*Pseudomonas sp.* in the *P. mandelii* SG
*P. lutea* SG							
100	PgKB30	*P. graminis*	GCF_013201545.1	Ps_E sp900585815	true	5	Ps_E sp900585815
160	PDD-13b-3	*P. graminis*	GCF_002093745.2	Ps_E graminis_C	true	3	Ps_E graminis_C
101	WP8	*P. graminis*	GCF_004364335.1	Ps_E graminis_D	true	1	Ps_E graminis_D
**157**	**P2653T**	***P. petrae***	**GCF_021728295.1**	**Ps_E sp021728295**	**true**	**4**	***P. petrae***
102	UASWS1507	*P. graminis*	GCF_001705435.1	Ps_E graminis_B	true	2	Ps_E graminis_B
**152**	**DSM11363T**	***P. graminis***	**GCF_900111735.1**	**Ps_E graminis**	**true**	**1**	***P. graminis***
**151**	**DSM 17257T**	***P. lutea***	**GCF_000759445.1**	**Ps_E lutea**	**true**	**2**	***P. lutea***
104	NFR16	*Pseudomonas sp.*	GCF_900108875.1	Ps_E sp900108875	true	1	P_E sp900108875
107	LS-2	*Pseudomonas* sp.	GCF_003605735.1	Ps_E sp003605735	true	1	Ps_E sp003605735
108	Bc-h	*Pseudomonas* sp.	GCF_002080045.1	Ps_E sp002080045	true	1	Ps_E sp002080045
109	NFACC02	*Pseudomonas* sp.	GCF_900110765.1	Ps_E sp900110765	true	1	Ps_E sp900110765
**110**	**ATCC 700689T**	***P. abietaniphila***	**GCF_900100795.1**	**Ps_E abietaniphila**	**true**	**1**	***P. abietaniphila***
105	LP_7_YM	*Pseudomonas* sp.	GCF_004364435.1	Ps_E sp004364435	true	1	Ps_E sp004364435
**106**	**IA19T**	***P. bohemica***	**GCF_002934685.1**	**Ps_E bohemica**	**true**	**1**	***P. bohemica***
103	RIT 409	*Pseudomonas* sp.	GCF_003052515.2	Ps_E sp003052515	true	2	Ps_E sp003052515
*P. cichorii* branch							
**115**	**DSM 50259T**	***P. cichorii***	**GCF_018343775.1**	**Ps_E cichorii**	**true**	**13**	***P. cichorii***
116	LJ1	*P. lijiangensis*	GCF_019718915.1	Ps_E cichorii_C	false	23	*P.lijiangensis*
**117**	**LJ2T**	***P. lijiangensis***	**GCF_018968705.1**	**Ps_E cichorii_C**	**true**	**23**	***P. lijiangensis***
118	Pc19-3	*P. capsici*	GCF_017165745.1	Ps_E capsici	false	16	*P. capsici*
**119**	**Pc19-1T**	***P. capsici***	**GCF_017165765.1**	**Ps_E capsici**	**true**	**16**	***P. capsici***
122	ICMP 3353	*P. cichorii*	GCF_003700275.1	Ps_E cichorii_B	true	1	Ps_E cichorii_B
*P. syringae* branch							
**123**	**LMG 26898T**	***P. asturiensis***	**GCF_900143095.1**	**Ps_E asturiensis**	**true**	**2**	***P. asturiensis***
**124**	**DOAB 1067T**	***P. triticumensis***	**GCF_014358015.1**	**Ps_E triticumensis**	**true**	**3**	***P. triticifolii***
125	ICMP 8820	*P. viridiflava*	GCF_002723575.1	Ps_E alliivorans	false	7	*P. alliivorans*
**126**	**20GA0068T**	***P. alliivorans***	**GCF_017826695.1**	**Ps_E alliivorans**	**true**	**7**	***P. alliivorans***
**127**	**ICMP 2848T**	***P. viridiflava***	**GCF_001642795.1**	**Ps_E viridiflava**	**true**	**1390**	***P. viridiflava***
128	ICMP 19473	*P. viridiflava*	GCF_003702045.1	Ps_E viridiflava_C	true	3	Ps_E viridiflava_C
**129**	**GEV388T**	***P. viridiflava***	**GCF_002087235.1**	**Ps_E floridensis**	**true**	**1**	***P. floridensis***
**130**	**BPIC 631T**	***P. avellanae***	**GCF_000444135.1**	**Ps_E avellanae**	**true**	**353**	***P. avellanae***
148	DC3000	*P. syringae* pv. *tomato*	GCF_000007805.1	Ps_E avellanae	false	353	*P. syringae* pv. *tomato*
**133**	**ICMP 2823T**	***P. cannabina***	**GCF_900100365.1**	**Ps_E cannabina**	**true**	**17**	***P. cannabina***
134	0788_9	*P. syringae* pv. *cilantro*	GCF_001293775.1	Ps_E syringae_P	true	3	Ps_E syringae_P
149	ICMP 12471	*P. syringae* pv. *coriandricola*	GCF_001400185.1	Ps_E syringae_P	false	3	Ps_E syringae_P
135	CC1583	*P. syringae*	GCF_000452665.1	Ps_E syringae_Q	false	41	Ps_E. syringae_Q
136	PD2766	*P. syringae* pv. *syringae*	GCF_001466965.1	Ps_E syringae_Q	false	41	Ps_E syringae_Q
159	PA-2-9E	*P. syringae*	GCF_023278085.1	Ps_E syringae_Q	true	41	Ps_E syringae_Q
137	CC1557	*P. syringae*	GCF_000452705.1	Ps_E syringae_F	true	2	Ps_E syringae_F
**131**	**ICMP 9151T**	***P. tremae***	**GCF_001401155.1**	**Ps_E tremae**	**true**	**85**	***P. tremae***
153	1_6	*P. coronafaciens* pv. *oryzae*	GCF_000156995.2	Ps_E tremae	false	85	*P. tremae/P. coronafaciens*
150	ICMP 8961	*P. coronafaciens* pv. *porri*	GCF_001400915.1	Ps_E tremae	false	85	*P. tremae/P. coronafaciens*
132	PDD-32b-74	*P. syringae*	GCF_002157375.1	Ps_E graminis_C	false	3	*Pseudomonas sp.*
**138**	**ICMP6289T**	***P. meliae***	**GCF_001400515.1**	**Ps_E amygdali**	**false**	**262**	***P. amygdali***
**139**	**ICMP4352T**	***P. savastanoi*** **pv. *savastanoi***	**GCF_001401285.1**	**Ps_E amygdali**	**false**	**262**	***P. amygdali***
**140**	**ICMP3918T**	***P. amygdali***	**GCF_002699855.1**	**Ps_E amygdali**	**true**	**262**	***P. amygdali***
**141**	**ICMP7848T**	***P. ficuserectae***	**GCF_001400815.1**	**Ps_E amygdali**	**false**	**262**	***P. amygdali***
**142**	**ICMP2855T**	***P. caricapapayae***	**GCF_001400735.1**	**Ps_E caricapapayae**	**true**	**13**	***P. caricapapayae***
144	B728a	*P. syringae* pv. *syringae*	GCF_000012245.1	Ps_E syringae_M	false	128	P. syringae_M
145	KUIN-1	*Pseudomonas* sp.	GCF_009176725.1	Ps_E syringae_M	true	128	P. syringae_M
**146**	**KCTC 12500T**	***P. syringae***	**GCF_000507185.2**	**Ps_E syringae**	**true**	**173**	***P. syringae***
**143**	**58T**	***P. cerasi***	**GCF_900074915.1**	**Ps_E cerasi**	**true**	**73**	***P. cerasi***
**147**	**DSM 14939T**	***P. congelans***	**GCF_900103225.1**	**Ps_E congelans**	**true**	**21**	***P. congelans***
No assigned branch							
9	v388	*Pseudomonas* sp.	GCF_003935425.1	Ps_E sp003935425	true	1	
10	JDS28PS106	*Pseudomonas* sp.		not available	--	--	*Pseudomonas* sp.
*P. caspiana* branch							
11	GR12-2	*P. syringae*	GCF_001698815.1	Ps_E syringae_A	true	1	P. syringae_A
12	CFII64	*Pseudomonas* sp.	GCF_000416235.1	Ps_E sp000416235	true	2	Ps_E sp000416235
20	CEB003	*P. syringae*	GCF_000737235.1	Ps_E sp000416235	false	2	Ps_E sp000416235
13	GAW0119	*P. syringae*	GCA_000737245.1	Ps_E syringae_J	true	1	Ps_E syringae_J
**3**	**FBF102T**	***P. caspiana***	**GCF_002158995.1**	**Ps_E caspiana**	**true**	**1**	***P. caspiana***
5	Irchel 3A5	*P. syringae*	GCF_900187575.1	Ps_E foliumensis	false	2	*P. folii*
**4**	**DOAB 1069T**	***P. foliumensis***	**GCF_014357575.1**	**Ps_E foliumensis**	**true**	**2**	***P. folii***
6	UB246	*P. syringae*	GCF_000452865.1	Ps_E sp002699985	false	11	Ps_E sp002699985
**7**	**CDFA 553T**	***P. quasicaspiana***	**GCF_021147825.1**	**Ps_E sp002699985**	**false**	**11**	***P. quasicaspiana***
19	ICMP 23753	*‘P. phytophila’*	GCF_025643095.1	not available	--	--	*P. quasicaspiana*
8	ICMP 561	*Pseudomonas* sp.	GCF_002699985.1	Ps_E sp002699985	true	11	Ps_E sp002699985
17	CDFA 550	*Pseudomonas* sp.	GCF_021147785.1	Ps_E sp002699985	false	11	Ps_E sp002699985
18	CDFA611	*Pseudomonas* sp.	GCF_021147805.1	Ps_E sp002699985	false	11	Ps_E sp002699985
**2**	**S25T**	***Pseudomonas*** **sp.**	**GCF_022790535.1**	**Ps_E sp022790535**	**true**	**1**	***P. maioricensis***
1	p8.G8	*P. viridiflava*	GCF_900602065.1	Ps_E sp900602065	true	1	Ps_E sp900602065
*P. ovata* branch							
14	StFLB209	*Pseudomonas* sp.	GCF_000829415.1	Ps_E sp000829415	true	4	Ps_E sp000829415
21	LJDD11	*Pseudomonas* sp.	GCF_024584215.1	Ps_E sp000829415	false	4	Ps_E sp000829415
23	21LCFQ02	*Pseudomonas* sp.	GCF_024129895.1	Ps_E sp000829415	false	4	Ps_E sp000829415
22	21LCFQ010	*Pseudomonas* sp.	GCF_024129905.1	Ps_E sp000829415	false	4	Ps_E sp000829415
**15**	**F51T**	***P. ovata***	**GCF_003131185.1**	**Ps_E ovata**	**true**	**3**	***P. ovata***
16	Leaf127	*Pseudomonas* sp.	GCF_001423155.1	Ps_E ovata	false	3	*P. ovata*

* In the GTDB taxonomy a representative genome is selected in each species cluster (true: genome representative of the species cluster; false: genome not representative of the species cluster).

**Table 2:** Phylogenomic identification of the *P. viridiflava* strains studied by Karasov et al. [33] that were assigned to a different species in the present study.

Strain	Genome Accession	GTDB Nomenclature	Genomes in GTDB Species Cluster	Genomes in Bioproject PRJEB24450	Assignation to Phylogenetic Group	Identification
p2.B6	GCA_900589175.1	Ps_E asturiensis	2	1	syringae G	*P. asturiensis*
p11.A4	GCA_900576645.1	Ps_e atacamensis	60	21	fluorescens G	*P. atacamensis*
p11.G1	GCA_900580895.1	Ps_E avellanae	353	20	syringae G	*P. avellanae/’P. tomato’*
p11.H3	GCA_900581025.1	Ps_E baltica	7	1	*P. rhizospherae/* *P. coleopterorum*	*P. baltica*
p11.B7	GCA_900580485.1	Ps_E canadensis	13	9	fluorescens G	*P. canadensis*
p11.C6	GCA_900580595.1	Ps_E coleopterorum	9	2	*P. rhizospherae/* *P. coleopterorum*	*P. coleopterorum*
p13.B5	GCA_900581935.1	Ps_E congelans	21	7	syringae G	*P. congelans*
p24.A10	GCA_900576715.1	Ps_E gregormendelii	10	6	fluorescens G	*P. gregormendelii*
p4.F8	GCA_900591125.1	Ps_E lurida	28	5	fluorescens G	*P. lurida*
p2.E10	GCA_900589445.1	Ps_E marginalis	19	1	fluorescens G	*P. marginalis*
p2.D4	GCA_900589345.1	Ps_E orientalis_A	22	12	fluorescens G	Ps_E orientalis_A
p9.C4	GCA_900602385.1	Ps_E ovata	3	1	syringae G	*P. ovata*
p11.F1	GCA_900580855.1	Ps_E poae	12	2	fluorescens G	*P. poae*
p8.D4	GCA_900601745.1	Ps_E salomonii	16	5	fluorescens G	*P. salomonii*
p2.D10	GCA_900589385.1	Ps_E sivasensis	14	6	fluorescens G	*P. sivasensis*
p23.G3	GCA_900586135.1	Ps_E sp001297015	11	5	fluorescens G	Ps_E sp001297015
p7.A9	GCA_900600635.1	Ps_E sp002699985	11	2	caspiana SG	*P. quasicaspiana*
p2.G9	GCA_900589715.1	Ps_E sp002843605	7	6	fluorescens G	Ps_E sp002843605
p11.A6	GCA_900576665.1	Ps_E sp002979555	11	11	fluorescens G	Ps_E sp002979555
p9.H9	GCA_900573885.1	Ps_E sp900573885	2	2	putida-oleovorans G	Ps_E sp900573885
p11.D4	GCA_900580675.1	Ps_E sp900580675	1	1	fluorescens G	Ps_E sp900580675
p11.F9	GCA_900580865.1	Ps_E sp900580865	1	1	fluorescens G	Ps_E sp900580865
p11.H11	GCA_900581005.1	Ps_E sp900581005	1	1	fluorescens G	Ps_E sp900581005
p13.D5	GCA_900582195.1	Ps_E sp900582195	2	2	fluorescens G	Ps_E sp900582195
p13.G10	GCA_900582425.1	Ps_E sp900582625	4	4	putida- oleovorans G	Ps_E sp900582625
p3.E1	GCA_900590325.1	Ps_E sp900583165	8	7	fluorescens G	Ps_E sp900583165
p26.D9	GCA_900588365.1	Ps_E sp900585815	5	4	lutea SG	Ps_E sp900585815
p23.C6	GCA_900585905.1	Ps_E sp900585905	1	1	caspiana SG	Ps_E sp900585905
p1.E6	GCA_900583105.1	Ps_E sp900589395	8	3	syringae G	Ps_E sp900589395
p4.B4	GCA_900590755.1	Ps_E sp900590755	1	1	caspiana SG	Ps_E sp900590755
p4.G3	GCA_900591205.1	Ps_E sp900591205	1	1	fluorescens G	Ps_E sp900591205
p2.G1	GCA_900589655.1	Ps_E sp900596015	8	5	*P. ryzospherae*-*P. coleopterorum*	Ps_E sp900596015
p8.G2	GCA_900601905.1	Ps_E sp900601905	1	1	caspiana branch	Ps_E sp900601905
p8.G8	GCA_900602065.1	Ps_E sp900602065	1	1	caspiana branch	Ps_E sp900602065
p4.G2	GCA_900591195.1	Ps_E synxantha_A	16	1	fluorescens G	Ps_E synxantha_A
p26.C10	GCA_900588235.1	Ps_E syringae	163	2	syringae G	*P. syringae*
p4.D11	GCA_900590885.1	Ps_E viridiflava_D	1	3	fluorescens G	Ps_E viridiflava_D

**Table 3:** Differential physiological and biochemical characteristics of *Pseudomonas maioricensis* S25T and closely related *Pseudomonas* named species type strains in the *P. caspiana* branch. *P. syringae* is included as the reference species in the group. +, positive; w, weak; −, negative.

Characteristic	*P. maioricensis* S25T	*P. caspiana* FBF102T	*P. quasicaspiana* LMG 32434T	*P. folii* LMG 32142T	*P. syringae* ATCC 19310T
**API 20 NE**: Hydrolysis of gelatine	−	−	−	−	+
**BIOLOG GENIII tests:**					
Carbon source utilisation assays					
D-Sorbitol	−	+	+	−	+
Pectin	+	−	−	−	−
D-Galacturonic Acid	+	+	+	+	−
Methyl Pyruvate	+	+	+	+	−
D-Galactonic Acid lactone	+	+	+	+	−
D-Trehalose	+	−	−	−	−
Beta-Methyl-D-Glucoside	+	−	−	−	w
Myo-Inositol	−	−	+	−	+
L-arginine	+	+	+	+	−
D-Cellobiose	+	−	−	−	+
D-Salicin	+	−	−	−	−
L-Aspartic Acid	+	+	+	+	−
D-Glucuronic Acid	+	+	+	+	w
Gentiobiose	+	−	−	−	−
Acetoacetic Acid	+	−	−	−	−
Sucrose	+	−	−	−	+
L-Histidine	+	+	+	+	−
D-Malic Acid	+	+	+	+	−
Propionic Acid	+	−	+	w	+
D-Aspartic Acid	+	−	−	−	−
Quinic acid	+	+	+	+	−
L-Malic Acid	+	+	+	+	w
D-Serine	w	+	−	w	+
Formic Acid	+	+	+	+	-
**Chemical sensitivity assays** (growth):					
Nalidixic Acid	−	+	+	+	w
Guanidine HCl	+	+	+	+	−
Lithium Chloride	+	−	+	−	+
Sodium Butyrate	+	w	w	w	−
8% NaCl	+	+	w	w	−
Minocycline	+	w	−	−	−
Sodium Bromate	+	−	−	−	−

**Table 4:** Protologue. Description of *Pseudomonas maioricensis* sp. nov.

Species name	*Pseudomonas maioricensis*
Species etymology	*P. maioricensis* (mai.or.i.cen’sis. M.L. masc./fem. Adj. maioricensis, pertaining to the island of Mallorca, where the type strain of the species was isolated) sp. nov.
Species status	
Designation of the type strain	S25
Strain collection numbers	CCUG 69272, CECT 30911
16S rRNA gene accession number	OR891488
Alternative housekeeping genes	*rpoD* gene (OR900883)
Genome accession number	LOHG01 (GCF_022790535.1)
Genome status	Complete
Genome size (pb)	5.911.519 bp
GC mol %	57
Country of origin	Spain
Region of origin	Balearic Islands
Date of isolation	2010
Source of isolation	Agricultural soil
Sampling date	2010
Geographic location	Mallorca
Latitude and Longitude	39°38′11.8″ N, 2°38′50.1″ E
Growth medium, incubation conditions used for standard cultivation	Lysis broth (LB) at 30 °C
Gram stain	Negative
Cell shape Cell size (length or diameter)	Rod 1.9–2.5 µm long and 0.7–1.1 µm wide
Motility Colony morphology	Motile with one polar flagellum Round (1–1.5 mm of diameter) flat and beige coloured, regular margins and translucent
Temperature range for growth	4–30
Temperature optimum	30
pH range for growth NaCl range for growth	5–9 0–6%
Metabolism	Aerobic, strictly respiratory
BIOLOG GENIII positive tests	Alfa-d-glucose, pectin, Tween 40, d-mannose, d-mannitol, glycyl-L-proline, d-galacturonic acid, methyl pyruvate, gamma-amino-butyric acid, d-fructose, d-arabitol, L-alanine, d-galactonic acid lactone, alfa-hydroxy butyric acid, d-trehalose, beta-methyl-d-glucoside, d-galactose, L-arginine, d-gluconic acid, L-lactic acid, beta-hydroxy-d,L butyric acid, d-cellobiose, d-salicin, glycogen, L-aspartic acid, d-glucuronic acid, citric acid, alfa-keto-butyric acid, gentiobiose, d-fucose, L-glutamic acid, glucuronamide, alfa-keto glutaric acid, acetoacetic acid, sucrose, L-histidine, mucic acid, d-malic acid, propionic acid, L-rhamnose, d-aspartic acid, L-pyroglutamic acid, quinic acid, L-malic Acid, acetic acid, inosine, L-serine, d-saccharic acid, formic acid
Negative tests with BIOLOG GENIII	d-raffinose, d-sorbitol, gelatin, p-hydroxy-phenylacetic acid, dextrin, alfa-d-lactose, d-maltose, d-lactic acid methyl ester, myo-inositol, 3-methyl glucose, N-acetyl-d-glucosamine, d-glucose-6-PO4, N-acetyl-beta-d-mannosamine, L-fucose, d-fructose-6-PO4, turanose, N-acetyl-d-galactosamine, stachyose, N-acetyl-neuraminic acid, bromo-succinic acid
Positive tests with API 20NE	Hydrolysis of aesculin, assimilation of glucose, arabinose, mannose, mannitol, gluconate, caprate, adipate, malate, citrate
Negative tests with API 20NE	Assimilation of N-acetyl-d-glucosamine, maltose, adipate, phenylacetate; reduction of nitrate to nitrite; reduction of nitrite to N2; indole production; glucose fermentation; presence of arginine dihydrolase and urease
Energy metabolism	Chemoorganotrophic, strictly respiratory.
Oxidase	Positive
Catalase	Positive
Pigment production on King A	Positive
Major fatty acids of the type strain	C10:0 3-OH (3.0%), C12:0 (6.3%), C12:0 2-OH (2.7%), C12:0 3-OH (5.3%), C16:0 (24.6%), C18:0 (1.5%), summed feature 3 C16:1 w7c/C16:1 w6c (342%) and summed feature 8 C18:1 w7c/C18:1 w6c (18.8%). C17:0 cyclo was present in low amounts (1.9%)
Biosafety level	1
Habitat	Soil
Biotic relationship	Free-living
Known pathogenicity	None

## Data Availability

All data are presented in the manuscript. Further details are available on request from the corresponding authors.

## Supplementary Materials

The following supporting information can be downloaded at: https://www.mdpi.com/article/10.3390/microorganisms12030460/s1: Figure S1: Phylogenomic analyses of 44 selected strains in the *P. avellanae* GTDB species cluster. (A) Core genome phylogeny based on the concatenated sequences of 1902 orthologous genes (1,572,618 nt); (B) ANI values, (C) GGDC values. Red line indicates the species thresholds. DC3000 and BPIC 631T groups are indicated in different colours. Strain name and genome assembly number is indicated for each genome; Figure S2: Phylogenomic analysis of selected strains in the *P. tremae* GTDB species cluster. autoMLST phylogenetic tree based on 85 housekeeping gene sequences; Figure S3: Transmission electron microscopy of a flagellated cell of *P. maioricensis* S25T; Figure S4. Pathogenicity test of *P. maioricensis* S25T on citrus leaves. *P. caspiana* FBF102T was used as a positive control; Figure S5: Dendrogram of the main protein profiles of whole cells obtained by MALDI-TOF MS. Table S1: Results of the experimental DNA-DNA hybridisation of *P. maioricensis* S25T, *P. caspiana* and related strains; Table S2: Genes and protein-coding genes of type strains in the *P. caspiana* branch in public databases and those detected by the M1CR0B1AL1Z3R web server; Table S3: RAST annotation of the *P. maioricensis* S25T-exclusive genes; Table S4: Prophage regions detected by the PHASTER server in the type strain genomes of the *P. caspiana* phylogenetic branch; Table S5: Virulence genes and antimicrobial resistance genes found in *P. maioricensis* S25T and % identity with the first hit in the NCBI protein BLAST analysis; Table S6: Genomes of the five strains in the *P. caspiana* branch were analysed for the presence of antibiotic resistance genes in the CARD website; Table S7: Comparative analysis of the T6SS-I genes of S25T and *P. caspiana* FBF102T; Table S8: Physiological and biochemical characterisation of *P. maioricensis* S25T; Table S9: Major fatty acid composition of *P. maioricensis* S25T cells.
