# Supplementary material for: Genome-Based Taxonomy of Species in the Pseudomonas syringae and Pseudomonas lutea Phylogenetic Groups and Proposal of Pseudomonas maioricensis sp. nov., Isolated from Agricultural Soil
Source: Microorganisms. 2024 Feb 24;12(3):460. doi: 10.3390/microorganisms12030460 (PMC10974278; doi:10.3390/microorganisms12030460)
Supplement: Supplementary file 1 [file microorganisms-12-00460-s001.zip › Supplementary_Figures.pdf]

Supplementary Figures

Genome-based taxonomy of the *Pseudomonas syringae* and *Pseudomonas lutea* phylogenetic groups of species and proposal of *Pseudomonas maioricensis sp. nov.,* isolated from agricultural soil

Magdalena Mulet, Margarita Gomila, Antonio Busquets, David Sánchez, Jorge Lalucat[1]* and Elena García-Valdés[1]*

*Corresponding authors

Figure S1. Phylogenomic analyses of 44 selected strains in the *P. avellanae* GTDB species cluster. **(A)** core genome phylogeny based on the concatenated sequences of 1,902 orthologous genes (1,572,618 nt); (B) aggregated ANI values, (C) GGDC values. Red line indicates the species thresholds. DC3000 and BPIC 631T groups are indicated in different colors. Strain name and genome assembly number is indicated for each genome.

Figure S2. Phylogenomic analysis of selected strains in the *P. tremae* GTDB species cluster. autoMLST phylogenetic tree based on 85 housekeeping gene sequences. Bar indicates phylogenetic distance.

Figure S3. Transmission electron microscopy of a flagellated cell of *P. maioricensis* S25T

Figure S4. Pathogenicity test of *P. maioricensis* S25^{T} on citrus leaves. *P. caspiana* FBF102T is the positive control.

Figure S5. Dendrogram of the main protein profiles of whole cells obtained by MALDI-TOF MS

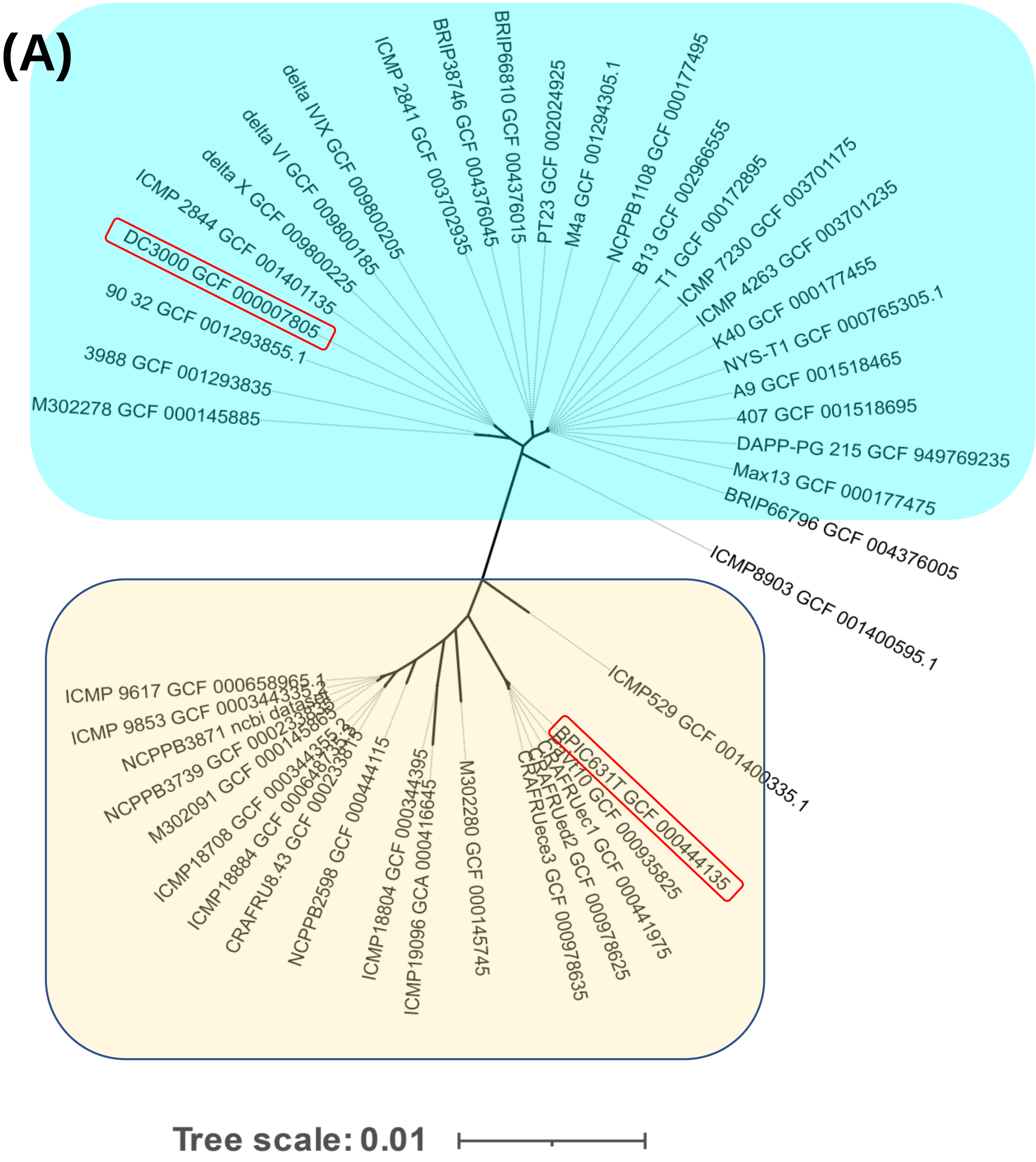

Figure S1. Phylogenomic analyses of 44 selected strains in the *P. avellanae* GTDB species cluster. **(A)** core genome phylogeny based on the concatenated sequences of 1,902 orthologous genes (1,572,618 nt); (B) aggregated ANI values, (C) GGDC values. Red line indicates the species thresholds. DC3000 and BPIC 631T groups are indicated in different colors. Strain name and genome assembly number is indicated for each genome.

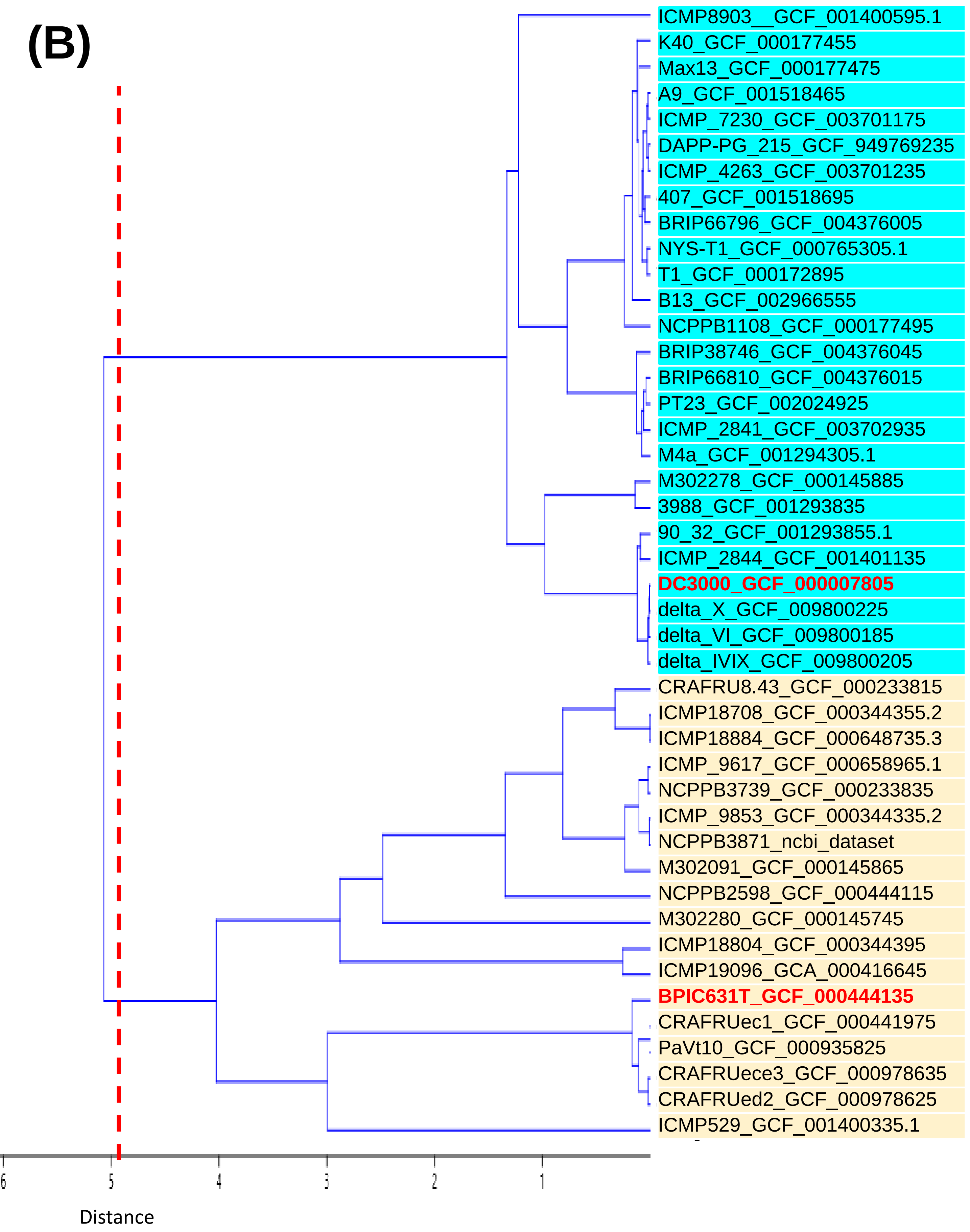

Figure S1. Phylogenomic analyses of 44 selected strains in the *P. avellanae* GTDB species cluster. (A) core genome phylogeny based on the concatenated sequences of 1,902 orthologous genes (1,572,618 nt); **(B)** aggregated ANI values, (C) GGDC values. DC3000 and BPIC 631T groups are indicated in different colors. Red line indicates the species thresholds.

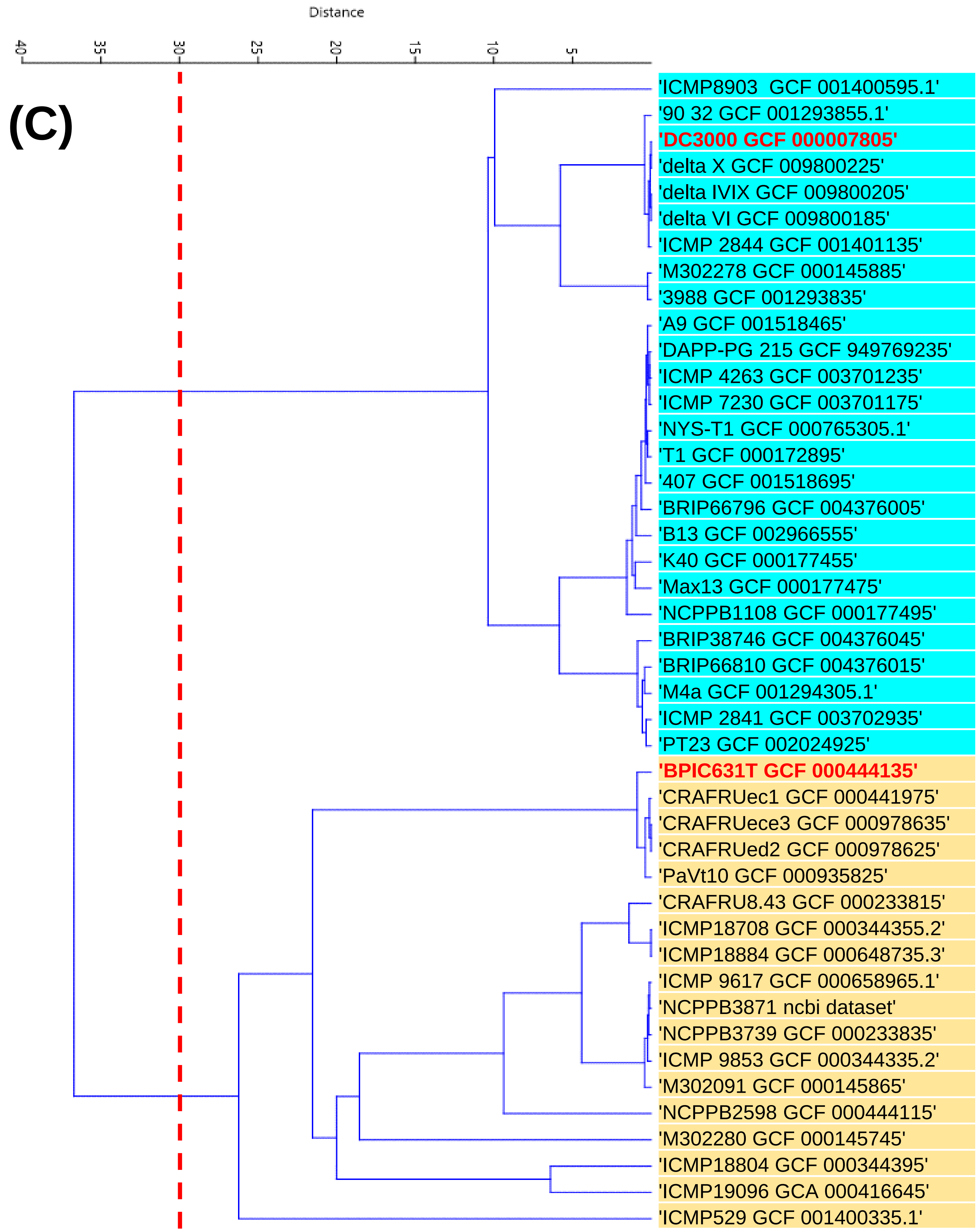

Figure S1. Phylogenomic analyses of 44 selected strains in the *P. avellanae* GTDB species cluster. (A) core genome phylogeny based on the concatenated sequences of 1,902 orthologous genes (1,572,618 nt); **(B)** aggregated ANI values, (C) GGDC values. DC3000 and BPIC 631T groups are indicated in different colors. Red line indicates the species thresholds.

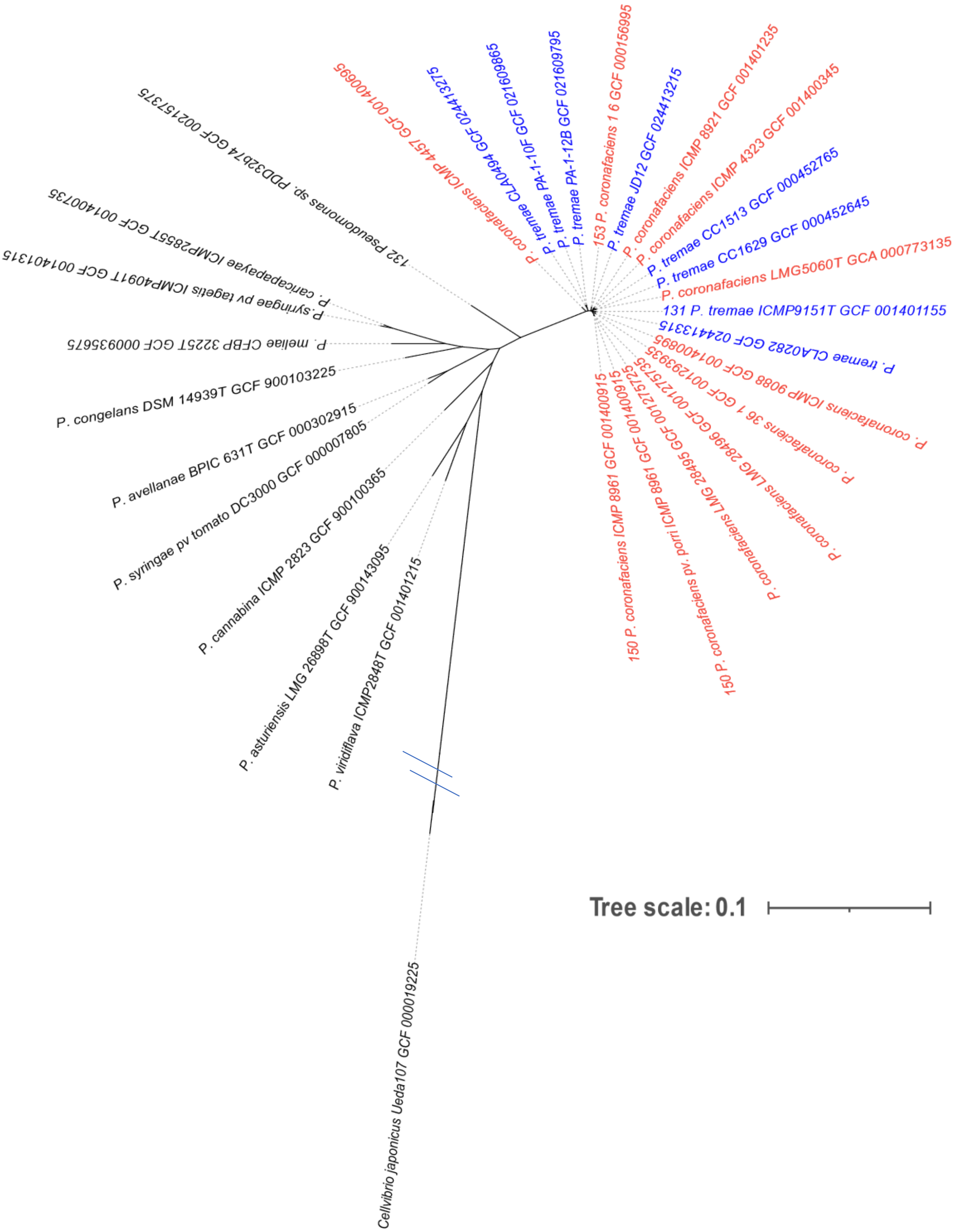

Figure S2. Phylogenomic analysis of selected strains in the *P. tremae* GTDB species cluster. autoMLST phylogenetic tree based on 85 housekeeping gene sequences. Bar indicates phylogenetic distance.

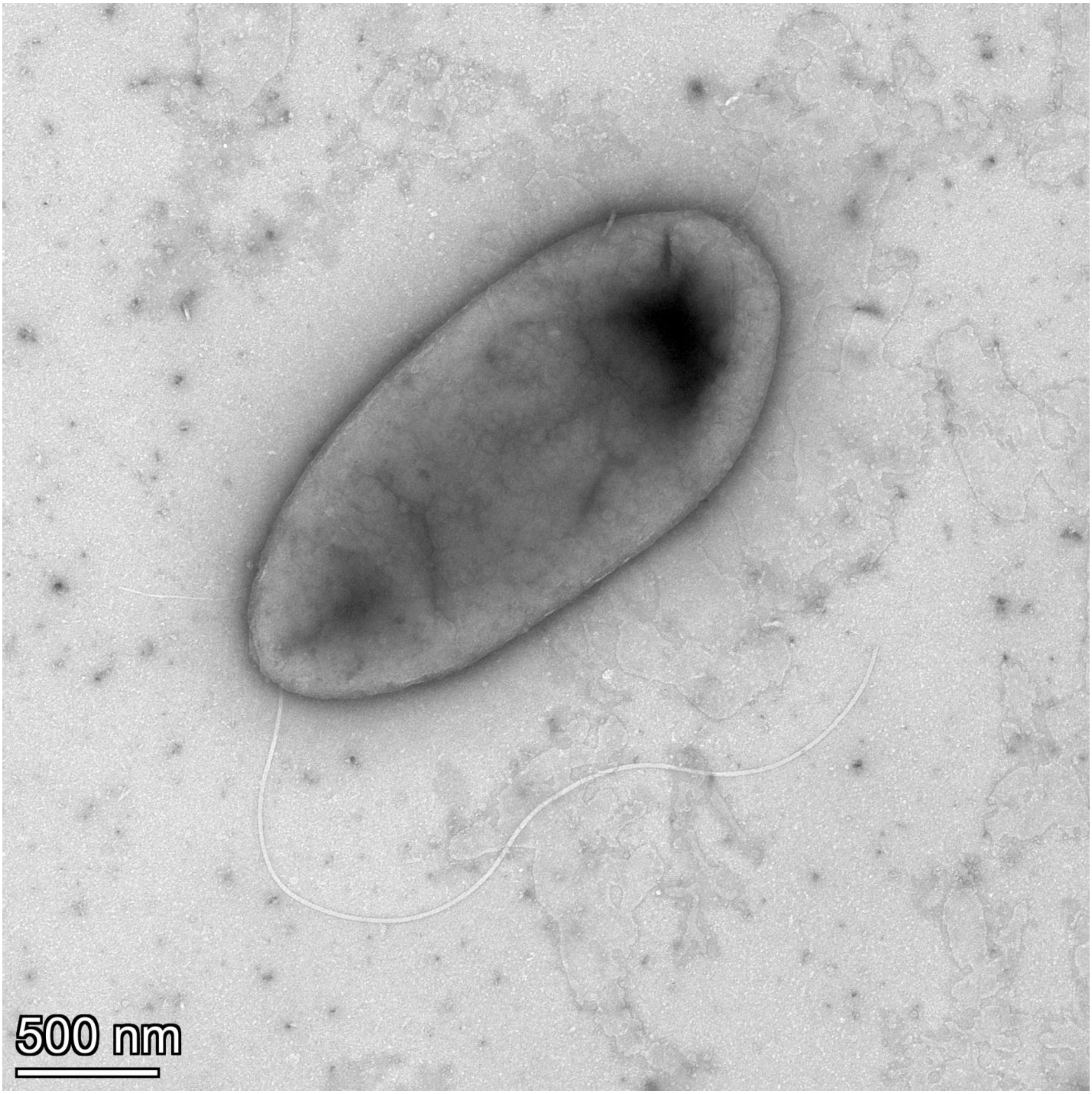

Figure S3. Transmission electron microscopy of a flagellated cell of *P. maioricensis* S25T.

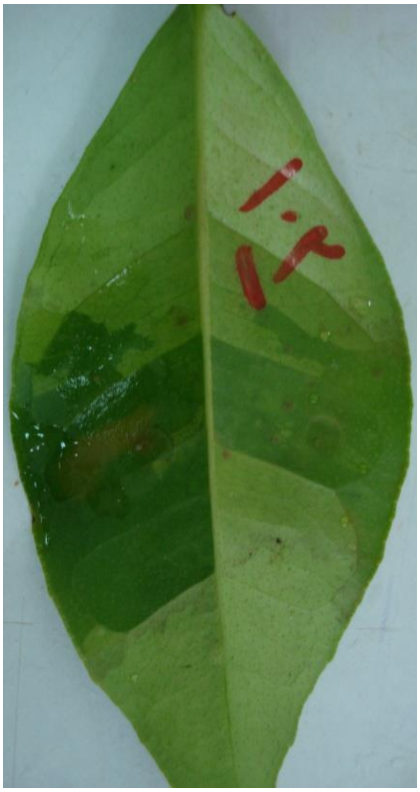

FBF102^{T} (Day 4)

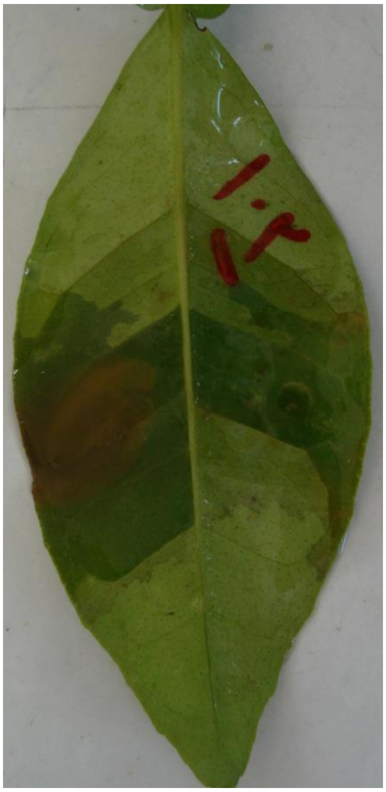

FBF102^{T} (Day 8)

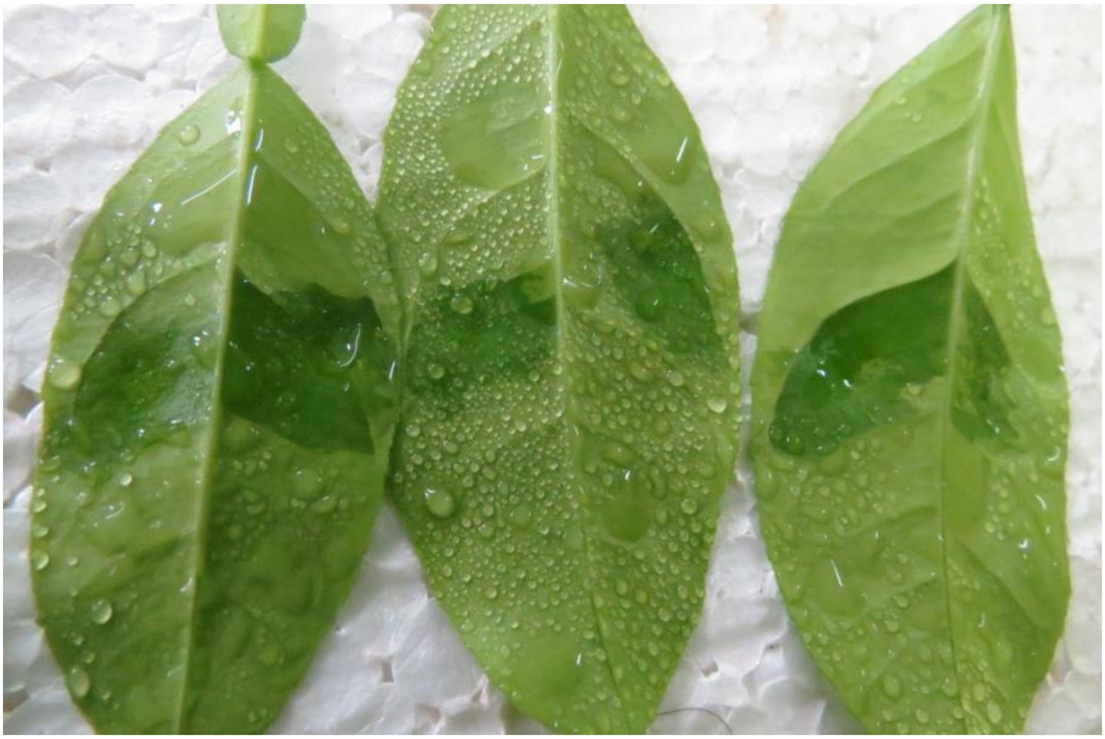

S25 (Day 7)

Figure S4. Pathogenicity test of *P. maioricensis* S25^{T} on citrus leaves. *P. caspiana* FBF102T is the positive control.

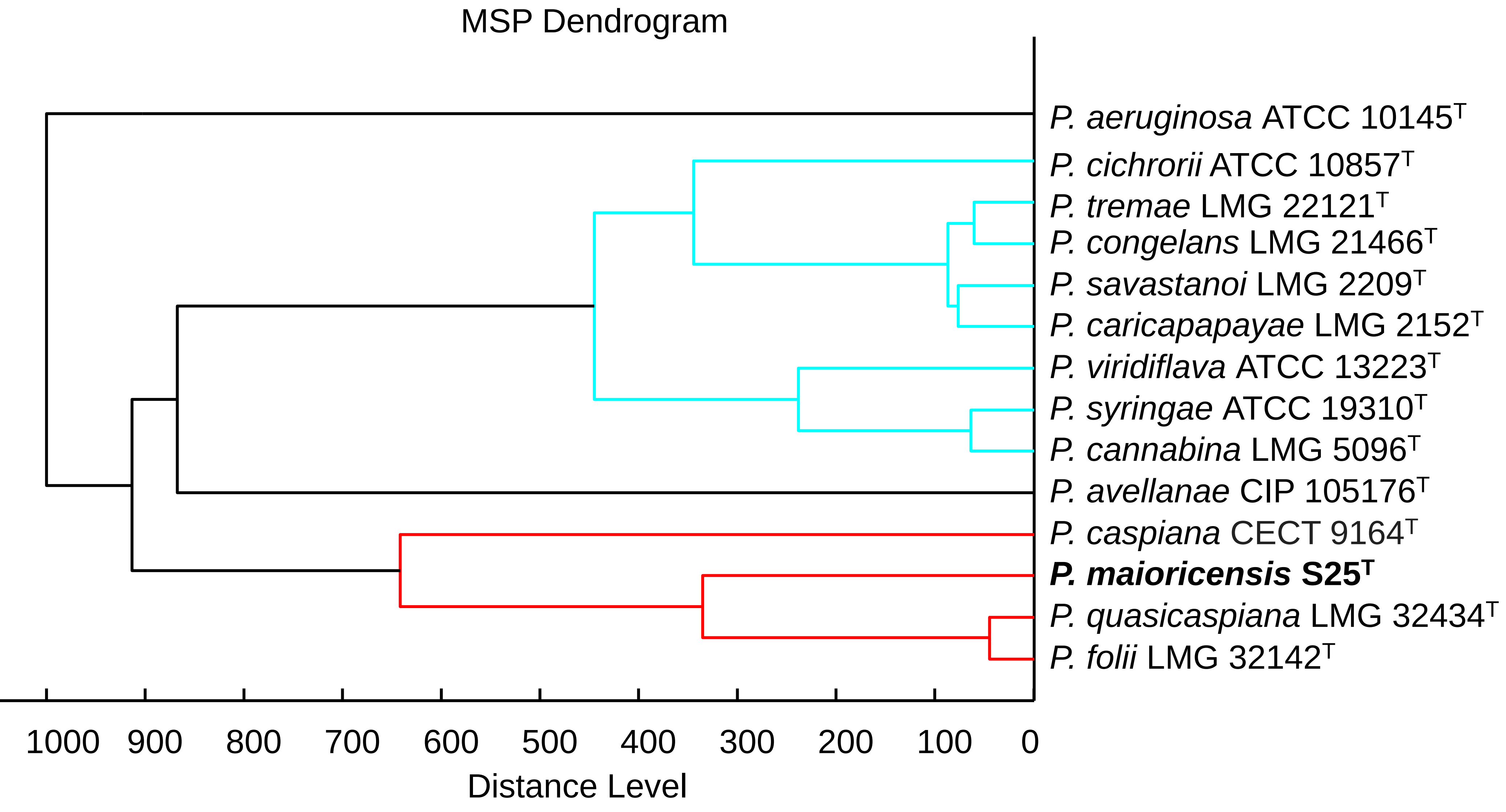

Figure S5. Dendrogram of the main protein profiles of whole cells obtained by MALDI-TOF MS.
